# Is it more effective for anhedonia and avolition? A systematic review and meta‐analysis of non‐invasive brain stimulation interventions for negative symptoms in schizophrenia

**DOI:** 10.1111/cns.14645

**Published:** 2024-03-03

**Authors:** Yuying Chen, Zhuofeng Li, Chao Yan, Laiquan Zou

**Affiliations:** ^1^ Chemical Senses and Mental Health Lab, Department of Psychology, School of Public Health Southern Medical University Guangzhou Guangdong China; ^2^ Key Laboratory of Brain Functional Genomics (MOE&STCSM), School of Psychology and Cognitive Science East China Normal University Shanghai China; ^3^ Department of Psychiatry, Zhujiang Hospital Southern Medical University Guangzhou Guangdong China

**Keywords:** anhedonia, meta‐analysis, schizophrenia, transcranial direct current stimulation, transcranial magnetic stimulation

## Abstract

**Background:**

Noninvasive brain stimulation (NIBS) techniques are a promising tool for treating the negative symptoms of schizophrenia. Growing evidence suggests that different dimensions of negative symptoms have partly distinct underlying pathophysiological mechanisms. Previous randomized controlled trials (RCTs) have shown inconsistent impacts of NIBS across dimensions.

**Objective:**

This systematic review and meta‐analysis evaluated the effects of NIBS on general negative symptoms, and on specific domains, including blunted affect, alogia, asociality, anhedonia, and avolition.

**Data Source**s**:**

PubMed, Web of Science, Embase, Cochrane CENTRAL, PsycINFO, OpenGrey, and Clinicaltrials.gov from the first date available to October, 2023.

**Results:**

Among 1049 studies, we identified eight high‐quality RCTs. NIBS significantly affects general negative symptoms (SMD = −0.54, 95% CI [−0.88, −0.21]) and all five domains (SMD = −0.32 to −0.63). Among dimensions, better effects have been shown for improvement of avolition (SMD = −0.47, 95% CI [−0.81, −0.13]) and anhedonia (SMD = −0.63, 95% CI [−0.98, −0.28]). Subgroup analyses of studies that applied once daily stimulation or >10 sessions showed significantly reduced negative symptom severity.

**Conclusion:**

NIBS exerts distinct effects across multiple dimensions of negative symptom, with treatment effects related to stimulation frequency and total sessions. These results need to be confirmed in dedicated studies.

## Graphical abstract

Our meta‐analysis revealed that noninvasive brain stimulation (NIBS) exerts distinct improvement effects across multiple dimensions of negative symptoms, with anhedonia and avolition being more effective. The treatment effect of NIBS may be related to the stimulation frequency and total sessions.

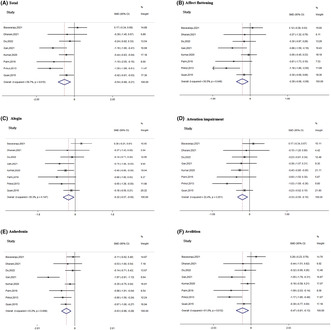

## 1 INTRODUCTION

Negative symptoms are core symptoms of schizophrenia, and clinical pharmacology therapy trials have shown poor efficacy in treating these symptoms.^1^, ^2^, ^3^, ^4^ Therefore, it is necessary to explore alternative treatment approaches for negative symptoms. Noninvasive brain stimulation (NIBS), including repetitive transcranial magnetic stimulation (rTMS) and transcranial direct current stimulation (tDCS), has emerged as a potential treatment modality for negative symptoms of schizophrenia because it is noninvasive, safe, and relatively convenient.^5^, ^6^, ^7^, ^8^ Several meta‐analyses investigating NIBS for improving negative symptoms have consistently demonstrated that patients receiving active stimulation show significantly better effects compared with sham groups, with average effect sizes ranging from 0.31 to 0.61.^7^, ^9^, ^10^

Recent and growing evidence suggests that different negative symptom dimensions have distinct underlying pathophysiological mechanisms.^11^, ^12^, ^13^ Apathy (asociality, anhedonia, and avolition) is related to impairments in motivational mechanisms, including reward anticipation, reward learning, and effort‐based decision‐making.^14^, ^15^, ^16^, ^17^ Task‐related functional magnetic resonance imaging (fMRI) studies have proven that ventral striatal (VS), ventromedial prefrontal cortex (VMPFC), orbitofrontal cortex (OFC), and dorsolateral prefrontal cortex (DLPFC) play key roles in the pathophysiology of apathy dimensions.^18^, ^19^, ^20^ Our understanding of the pathophysiology of diminished expression (blunted affect and alogia) is less well developed and has focused on three main areas: emotion expression deficits, dysfunction in emotion perception, and insufficient cognitive resources for speech production.^16^, ^17^, ^21^ Some research show a potential role of the anterior cingulate cortex (ACC), amygdala (AMY), and ventrolateral prefrontal cortex (VLPFC) in these dimensions.^22^, ^23^, ^24^

Previous randomized controlled trials (RCTs) have shown that NIBS impacts each of these negative symptom dimensions differently.^25^, ^26^ When Kumar and colleagues^27^ applied 20 Hz rTMS to the DLPFC of patients with schizophrenia, the rTMS group had significantly lower anhedonia, alogia, and attention impairment subscale scores compared with the sham group. In Gan's study, patients treated with 10 Hz rTMS show significant improvements in effective flattening and anhedonia.^28^ Palm and colleagues^29^ did not find significantly improve anhedonia in patients with schizophrenia after 2 mA tDCS treatment. However, these studies varied in subjects, stimulus parameters, stimulus techniques, results, and other factors. Thus, whether NIBS is effective across negative symptom dimensions, and whether its effectiveness varies by dimension, is currently unknown.

This systematic review and meta‐analysis addresses the growing evidence that negative symptom dimensions have partly distinct underlying pathophysiological mechanisms, including from previous RCTs showing that NIBS impacts these dimensions differently. Current meta‐analyses in this field have focused on overall negative symptom improvements, neglecting dimension‐specific differences.^7^, ^9^, ^10^ Thus, we conducted a meta‐analysis of eight studies to examine the effects of NIBS on schizophrenia's negative symptoms with subgroup analyses.

## 2 METHODS

The methods are based on the Preferred Reporting Items for Systematic Reviews and Meta‐Analyses (PRISMA) (see Table S1).^30^ Two authors (Chen, Y. & Li, Z.) independently conducted the literature search, study selection, data extraction, and quality assessment. Disagreements were resolved by consensus, following joint examination of the studies. No registration information or review protocol for this review.

### 2.1 Literature search

We conducted a comprehensive literature search using online databases PubMed, Web of Science, Embase, Cochrane CENTRAL, PsycINFO, OpenGrey, and Clinicaltrials.gov, up to October 2023. We used the following search term strategy: non‐invasive brain stimulation AND negative symptoms AND schizophrenia. A detailed description of keywords and search results is available in Table S2. Reference lists of related articles and reviews were also screened. There was no restriction on publication date.

### 2.2 Study selection

All searched studies were imported into the Zotero software for screening. Subsequently, duplicate articles were removed and unpublished studies were excluded. For non‐English articles, we read the English abstracts or used a translator. The inclusion criteria for the current meta‐analysis were as follows: (1) RCTs; (2) studies that used NIBS to treat patients with schizophrenia; (3) trials with a minimum duration of one intervention week; and (4) trials that reported mean changes and their standard deviation (SD) for each negative symptom dimension throughout the trial for both the intervention and control groups, or provided the necessary information for effect size calculation. If data were unreported, the corresponding author was contacted via email to request necessary information. If more than one published article used a single dataset, the most comprehensive article was included.

### 2.3 Data extraction

The following data were extracted from included papers: first author, publication year, population, intervention, comparison, outcome, study design, country, and funding source(s) (Table 1).

**TABLE 1:** Participant characteristic and NIBS parameters of studies included.

Authors (Year)	Sample Size	Diagnosis	Diagnosis criteria	Sex (male %)	Age (mean ± SD)	Illness Duration (mean ± SD; years)	Comparison	Sessions per day	Total sessions	Treatment duration	Extracted outcome measures	Study design	Follow‐up (from the last treatment)	Country	Funding sources
Basavaraju (2021)^34^	30 30	SCZ	DSM‐V	80 73.33	31.17 ± 9.9 34.17 ± 8.06	11.47 ± 3.7 11.13 ± 3.9	iTBS at vermal part of cerebellum Sham control	2	10	5 days	SANS	RCT	5 weeks	India	Research Training Fellowship awarded by DBT/Wellcome Trust India Alliance
Dharani (2021)^33^	7 7	SCZ	ICD‐10	85.71	39.14 ± 3.76 33.85 ± 6.81	7.42 ± 6.26 7.57 ± 4.96	High definition 2 mA Anode tDCS at F3, cathode at AF3, F7, FC5, and FC1 Sham control	2	10	5 days	SANS	RCT	1 week	India	NA
Du (2022)^25^	25 22	SCZ	ICD‐10	48 50	45.9 ± 10.0 45.1 ± 10.4	≥ 5	10 Hz rTMS at left DLPFC (F3) Sham control	1	20	4 weeks	SANS	RCT	4 weeks	China	Key Diagnosis and Treatment Program of Suzhou, The Scientific and Technological Program of Suzhou, and Introduction Project of Suzhou Clinical Expert Team
Gan (2021)^28^	17 16	SCZ	DSM‐IV	70.59 62.50	35.70 ± 11.36 34.44 ± 12.50	16.54 ± 13.22 13.19 ± 9.66	10 Hz rTMS at DMPFC Sham control	1	20	4 weeks	SANS	RCT	4 weeks	China	National Natural Science Foundation of China, Key Program of Multidisciplinary Cross Research Foundation of Shanghai Jiao Tong University, Shanghai Municipal Health Commission Clinical Research Project, Key Program of Shanghai Mental Health Center Clinical Research Center, Shanghai Mental Health Center Project, Qihang project of Shanghai Mental Health Center, Shanghai Key Laboratory of Psychotic Disorders, and Shanghai Clinical Research Center for Mental Health
Kumar (2020)^27^	50 50	SCZ	ICD‐10	58 56	32.4 ± 9.20 30.8 ± 9.34	10.48 ± 6.37 8.46 ± 7.06	20 Hz rTMS at left DLPFC (F3) Sham control	1	20	4 weeks	SANS	RCT	4,8,12 and 16 weeks	India	Department of science and Technology under CSRI Initiative
Palm (2016)^29^	10 10	SCZ	DSM‐IV	50 100	38.4 ± 12.9 34.1 ± 10.7	7.1 ± 6.1 13.8 ± 12.1	2 mA Anode tDCS at F3, cathode at Fp2 Sham control	1	10	2 weeks	SANS	RCT	2 weeks	Germany	funded by FöFoLe grant 724 of the Ludwig Maximilian University to U.P. and more recently supported by the German Center for Brain Stimulation (GCBS) research consortium, funded by the Federal Ministry of Education and Research (BMBF)
Prikryl (2013)^26^	23 17	SCZ	ICD‐10	100 100	31.60 ± 8.04 33.94 ± 9.98	4.91 ± 5.09 5.89 ± 7.91	10 Hz rTMS at left DLPFC (F3) Sham control	1	15	3 weeks	SANS	RCT	/	Czech Republic	provided by the project “CEITEC — Central European Institute of Technology” from the European Regional Development Fund and by the project (Ministry of Health, Czech Republic) for conceptual development of research organization (University Hospital Brno, Brno, Czech Republic)
Quan (2015)^35^	78 39	SCZ	DSM‐IV	56.41 71.79	46.87 ± 7.87 46.87 ± 9.07	20.53 ± 10.97 17.97 ± 11.03	10 Hz rTMS at left DLPFC (F3) Sham control	1	20	6 weeks	SANS	RCT	4,12 and 24 weeks	China	Beijing Municipal Science & Technology Commission grant

*Note*: The first row for each study represents the active group and the second row represents the sham group.

Abbreviations: DSM‐IV, Diagnostic and Statistical Manual‐ IV; DSM‐V, Diagnostic and Statistical Manual‐V; ICD‐10, International Classification of Diseases‐10; iTBS, intermittent theta‐burst stimulation; NA, not available; rTMS, repetitive transcranial magnetic stimulation; SCZ, patients with schizophrenia; tDCS, transcranial direct current stimulation.

### 2.4 Statistical analysis

This meta‐analysis was conducted using Stata 15 (Stat Corp., College Station, Texas, USA). Scale differences between pre‐ and post‐treatment (mean and SD) in the intervention and sham groups were used to calculate the overall effect sizes. If SD values for the mean change were unavailable and the correlation coefficients were unreported, post‐treatment scores and SD were used in accordance with the Cochrane Handbook for Systematic Review of Intervention guidelines (http://www.cochrane‐handbook.org). Standardized mean differences (SMD) for each study were calculated for analyses. A total SMD was calculated for each negative symptom dimension. Positive SMDs were interpreted as indicating a favorable effect of sham stimulation, while negative SMDs were interpreted as indicating a favorable effect of active stimulation. To obtain overall effect sizes, we applied a random‐effects model that took study heterogeneity into account. We used chi‐square to assess the heterogeneity of the data. Two‐sided 95% confidence intervals (CI) were used to assess significance. Funnel plots and Egger regression were used to evaluate potential publication bias. Sensitivity analysis was examined using the Rosenthal fail‐safe N‐test.^31^ The fail‐safe number is computed as the number of studies with an average sample size and nonsignificant outcomes that are needed in order for the effect size of the meta‐analysis to reach nonsignificance. Upon identifying substantial heterogeneity, we conducted subgroup analyses using a random‐effects model.

We used the Physiotherapy Evidence Database (PEDro) scale to report quality assessment (Table S3). The PEDro scale consists of 11 items encompassing external validity (item 1), internal validity (items 2–9), and statistical reporting (items 10 and 11): (1) Eligibility criteria and source; (2) Random allocation; (3) Concealed allocation; (4) Baseline comparability; (5) Subjects blinded; (6) Therapists blinded; (7) Raters blinded; (8) Adequate follow‐up (>85%); (9) Intention‐to‐treat analysis; (10) Between‐group statistical comparisons; and (11) Reporting of point measures and measures of variability.^32^ We followed the Cochrane Handbook for GRADE (Grading of Recommendations, Assessment, Development, and Evaluations) for quality assessment. We use the GRADEpro GDT (https://gradepro.org/) online platform to evaluate the evidence quality of the results and manually create an evidence summary table.

## 3 RESULTS

**FIGURE 1:**
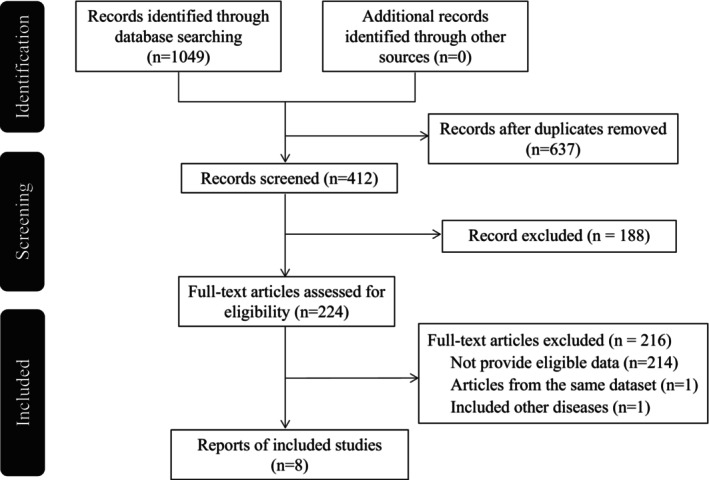
Preferred Reporting Items for Systematic Reviews and Meta‐Analyses (PRISMA) flow diagram of included/excluded studies.

A total of 224 articles were considered for a full‐text review, and eight articles were included in the meta‐analysis (Figure 1). The primary outcomes of meta‐analyses showed significant effects of NIBS on general negative symptoms and five dimensions. Among the five dimensions, better improvement effects were found on avolition and anhedonia. The secondary outcome was a subgroup analysis of the included studies based on stimulation frequency and total sessions.

### 3.1 Included studies

Figure 1 illustrates the PRISMA flow diagram of the inclusion and exclusion process. The literature search identified eight eligible studies (one for iTBS, two for tDCS, and five for rTMS).^25^, ^26^, ^27^, ^28^, ^29^, ^33^, ^34^, ^35^ The participant characteristics and NIBS parameters for each included study are in Table 1. A cumulative sample of 431 patients with schizophrenia was included across all studies. Their mean age was 38.15 ± 11.38 years, and the mean (range) proportion of male participants was 66.59% (48%–100%). All included RCTs allowed concurrent treatment with antipsychotics during the study period. Of the included studies, one targeted the dorsal medial prefrontal cortex (DMPFC),^28^ one targeted the cerebellar vermis area VII‐B,^34^ and the other six typically targeted the DLPFC.^25^, ^26^, ^27^, ^29^, ^33^, ^35^ Two studies administered two daily sessions,^33^, ^34^ while the other six administered one daily session.^25^, ^26^, ^27^, ^28^, ^29^, ^35^ Three studies administered NIBS for 10 stimulation sessions,^29^, ^33^, ^34^ and the other five studies applied a total of 15 or 20 stimulation sessions.^25^, ^26^, ^27^, ^28^, ^35^

Quality assessment details are summarized in Table S3. Based on the PEDro scores, we found no concerns regarding the quality of the included studies and the subsequent results.

### 3.2 Primary outcomes

**FIGURE 2:**
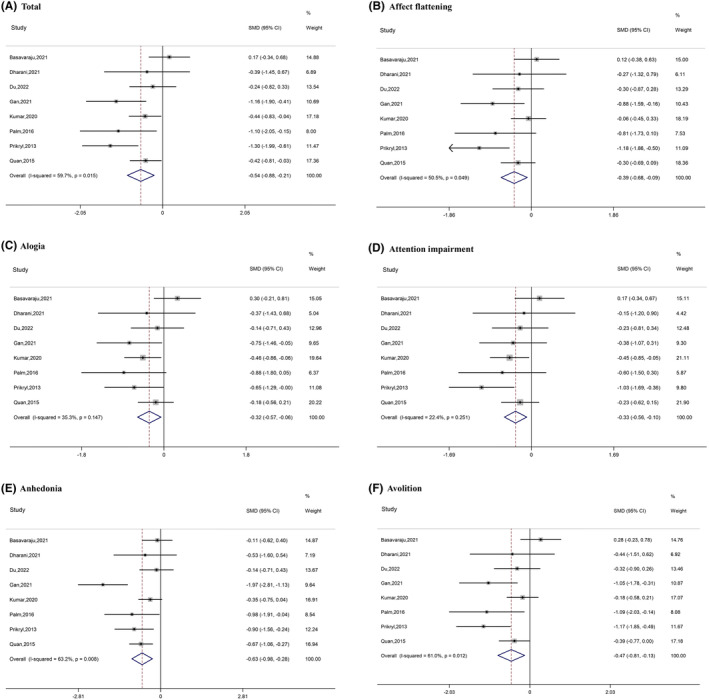
Forest plots of the results of the meta‐analyses.

The overall effects of NIBS efficacy on the negative symptoms of schizophrenia were statistically significant (Figure 2A–F). Total Scale for the Assessment of Negative Symptoms (SANS) scores were significantly reduced after tDCS or rTMS compared with the sham condition (SMD = −0.54, 95% CI [−0.88, −0.21]) (Figure 2A), indicating significantly greater symptom reduction from active treatment relative to sham. Publication bias was examined; visual inspection of the funnel plot and Egger's test (*t* = −1.29, *p* = 0.246) indicated that publication bias was not significant (Figure S1a). The Rosenthal fail−safe N suggested that 81 studies with null findings would reduce the overall‐effect *p*‐value to become nonsignificant.

Among the negative symptom dimensions, all five subscales were significantly reduced after NIBS compared with the sham condition: affect flattening (SMD = −0.39, 95% CI [−0.68, −0.09]); alogia (SMD = −0.32, 95% CI [−0.57, −0.06]); attention impairment (SMD = −0.33, 95% CI [−0.56, −0.10]); anhedonia (SMD = −0.63, 95% CI [−0.98, −0.28]); and avolition (SMD = −0.47, 95% CI [−0.81, −0.13]) (Figure 2B–F). Publication bias was examined; visual inspection of the funnel plot and Egger's test suggested that publication bias was not significant: affect flattening (*t* = −1.66, *p* = 0.148); alogia (*t* = −1.02, *p* = 0.348); attention impairment (*t* = −0.55, *p* = 0.604); anhedonia (*t* = −1.01, *p* = 0.353); and avolition (*t* = −1.60, *p* = 0.160) (Figure S1b–f). The Rosenthal fail‐safe N suggested that more than 29 studies with null findings would reduce the overall *p*‐value to nonsignificance (affect flattening: 41; alogia: 29; attention impairment: 30; anhedonia: 113; avolition: 58).

### 3.3 Secondary outcome

**FIGURE 3:**
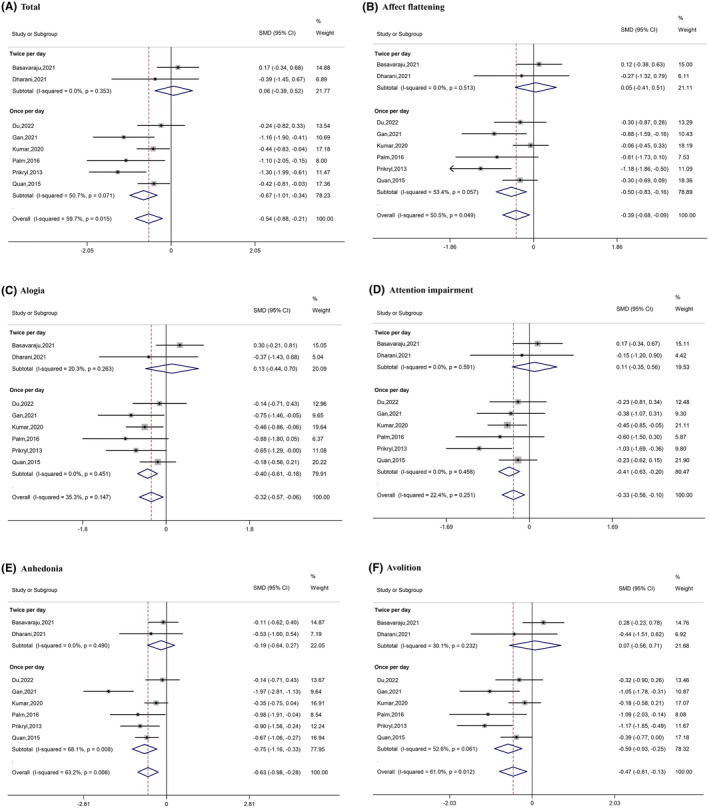
Forest plots of the results of the subgroup analysis (based on frequency).

Subgroup analysis was conducted based on stimulation frequency (once or twice daily) and total sessions (10 or >10). Subgroup analysis of studies with the lower stimulation frequency (once daily) revealed a significant difference in therapeutic effects between active and sham stimulation: total (SMD = −0.67, 95% CI [−1.01, −0.34]); affect flattening (SMD = −0.50, 95% CI [−0.83, −0.16]); alogia (SMD = −0.40, 95% CI [−0.61, −0.18]); attention impairment (SMD = −0.41, 95% CI [−0.63, −0.20]); anhedonia (SMD = −0.75, 95% CI [−1.16, −0.33]); and avolition (SMD = −0.59, 95% CI [−0.93, −0.25]). Of note, the effects for this group (once daily) are higher than the general findings. However, studies with the higher stimulation frequency (twice daily) did not show a significant difference between active and sham groups on either the total score or any subscale score (Figure 3A–F).

**FIGURE 4:**
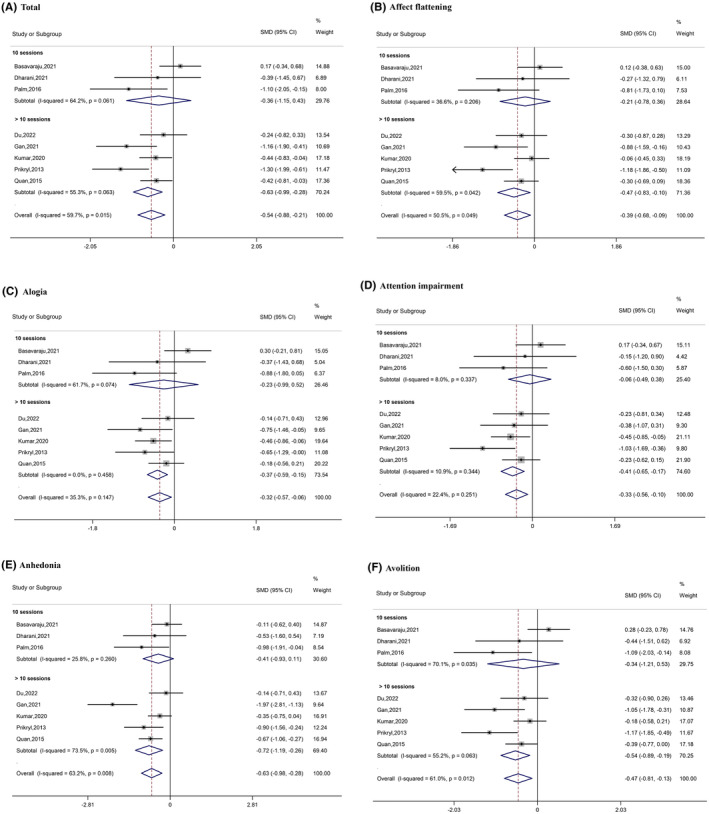
Forest plots of the results of the subgroup analysis (based on total sessions).

Subgroup analysis of studies that administered >10 sessions revealed a significant difference in therapeutic effects between active and sham stimulation: total (SMD = −0.63, 95% CI [−0.99, −0.28]); affect flattening (SMD = −0.47, 95% CI [−0.83, −0.10]); alogia (SMD = −0.37, 95% CI [−0.59, −0.15]); attention impairment (SMD = −0.41, 95% CI [−0.65, −0.17]); anhedonia (SMD = −0.72, 95% CI [−1.19, −0.26]); and avolition (SMD = −0.54, 95% CI [−0.89, −0.19]). However, studies including only 10 sessions did not show a significant difference between the active and sham groups on either total score or any subscale score (Figure 4A–F).

### 3.4 GRADE system recommendation grading

This meta‐analysis had six main outcomes: NIBS impacts on general negative symptoms and each of five dimensions (affect flattening, alogia, attention impairment, anhedonia, and avolition) among patients with schizophrenia. The GRADE system‐based recommended grading for each outcome is presented in Table S4. Among them, four outcomes are considered of low quality, and two results are of moderate quality.

## 4 DISCUSSION

Significant recent progress has been made regarding the effectiveness of NIBS for treating patients with schizophrenia, as evidenced by findings from basic and clinical studies. Several meta‐analyses have consistently demonstrated the positive therapeutic effects of NIBS on the negative symptoms of schizophrenia.^7^, ^9^, ^10^, ^36^ However, these studies are limited insofar as they often treat negative symptoms as a homogeneous construct, neglecting the diverse dimensions and unique characteristics within symptom domains. Herein, we summarized the impact of NIBS on negative symptoms, including five specific dimensions, to supplement the existing research.

The results of this meta‐analysis show that NIBS has different effects across the negative symptom dimensions. According to SMD interpretations (<0.4 is a small effect size, 0.4–0.7 is a moderate effect size, and >0.7 is a large effect size), NIBS intervention has a moderate effect size on improving avolition (SMD = −0.47) and anhedonia (SMD = −0.63), and a small but statistically significant effect on affect flattening (SMD = −0.39), attention impairment (SMD = −0.33), and alogia (SMD = −0.32). From the perspective of neural mechanisms, growing evidence shows that anhedonia and avolition are linked, and that both may derive from deficient reward network functioning.^11^ Further analysis of the neuroimaging findings in patients with schizophrenia has shown that brain regions related to anhedonia and avolition overlap with reward networks, including the VS, ACC, and OFC.^37^ In the reward system, information is delivered from VS and OFC to ACC, where cost/benefit analysis is run. In turn, the ACC sends projections to the anterior VMPFC and DLPFC for ultimate decision‐making.^37^, ^38^ The DLPFC directly affects relevant brain regions of the reward system, resulting in better therapeutic effects on avolition and anhedonia.^39^, ^40^, ^41^

Subgroup analysis indicated that >10 stimulation sessions are needed to reduce the negative symptoms of schizophrenia. The cumulative clinical effects of more sessions have also been reported in other rTMS and tDCS studies.^7^ In addition to avolition and anhedonia, affect flattening also showed a moderate effect in subgroup analysis but a small effect in the overall meta‐analysis. Therefore, we assume that the number of sessions is important to the effects of NIBS on the negative symptoms of schizophrenia. To the extent possible, future studies should evaluate >10 NIBS sessions to ensure therapeutic effectiveness.

The subgroup analysis also showed that studies in which stimulation was administered once daily led to a significant therapeutic difference between active and sham groups. In a five‐day study, tDCS induced greater increases in motor evoked potential amplitude when administered once daily compared with twice daily, consistent with the finding herein.^42^ In contrast, a subgroup analysis of studies administering NIBS more often than once daily revealed a significant difference in therapeutic effects between active tDCS and sham.^10^ Because only two studies fell into the twice‐daily stimulation category, this finding may not be robust. Though current trials investigating the therapeutic value of neuroplasticity‐inducing NIBS typically use a protocol of once daily administration for 1–2 weeks, there is no direct scientific evidence to support this as a best practice.^43^ Indeed, some researchers have asserted that this stimulation frequency is used for practical or convenience‐based reasons.^44^ Overall, more experimental data are needed to further refine the optimal parameters for NIBS treatment effects.

Several limitations of this systematic review and meta‐analysis should be noted. First, it was based on only eight studies, because few available studies include SANS subscale scores. We contacted corresponding authors by email whenever possible, but ultimately had to exclude studies that did not provide sufficient data. Researchers should consider including raw data or providing effect sizes whenever possible, to facilitate their contributions to future meta‐analyses. Although the number of included studies was relatively small, our results are nonetheless meaningful; on the basis of this evidence, future NIBS interventions can be tailored to individual patients’ symptom profiles, with protocols determined by their specific symptom dimensions. Second, only the effects of stimulation frequency and number of sessions were considered. However, in the Table 1 summary, we note that the included studies also differ by region, population, center setting, and other aspects that may affect treatment effectiveness. However, those data were insufficient for further subgroup analyses. The optimal NIBS parameters have yet to be determined. More research and additional, original data will be conducive to developing and standardizing a treatment manual and promoting the development of this technology. Third, the long‐term effects of NIBS were not explored herein because of variations in durations between the last stimulation visit and follow‐up, which either varied across studies or were unreported. Finally, although we searched the gray literature library, we were unable to avoid all deviations. According to GRADE, our evidence levels are low‐to‐moderate quality and thus require careful interpretation.

## 5 CONCLUSION

This systematic review and meta‐analysis show that there are significant effects of NIBS on general negative symptoms and on all five specific domains. Among the latter, there are more pronounced improvement effects on avolition and anhedonia. These cumulative results suggest that once daily and more than 10 stimulation sessions are likely needed to improve negative symptoms in patients with schizophrenia. Guidance standards on NIBS stimulation treatment parameters are needed for future research and clinical treatments.

## AUTHOR CONTRIBUTIONS

Conceptualization, Laiquan Zou; references selection and data extraction, Yuying Chen and Zhuofeng Li; meta‐analysis and original draft, Yuying Chen; review and editing, Chao Yan and Laiquan Zou. All authors have read and agreed to the published version of the manuscript.

## FUNDING INFORMATION

This work was supported by National Natural Science Foundation of China (82371561), and Guangzhou Municipal Science and Technology Project (2023A04J1966).

## CONFLICT OF INTEREST STATEMENT

The authors declare that they have no known competing financial interests or personal relationships that could have appeared to influence the work reported in this paper.

## Supporting information


Figure S1

Table S1–S4

## Data Availability

Data associated with this study has not been deposited into any publicly available repository. Data will be made available on request.
