# Supplementary material for: Is it more effective for anhedonia and avolition? A systematic review and meta‐analysis of non‐invasive brain stimulation interventions for negative symptoms in schizophrenia
Source: CNS Neurosci Ther. 2024 Mar 3;30(3):e14645. doi: 10.1111/cns.14645 (PMC10909625; doi:10.1111/cns.14645)
Supplement: Supplementary file 1 — Figure S1 [file CNS-30-e14645-s002.doc]

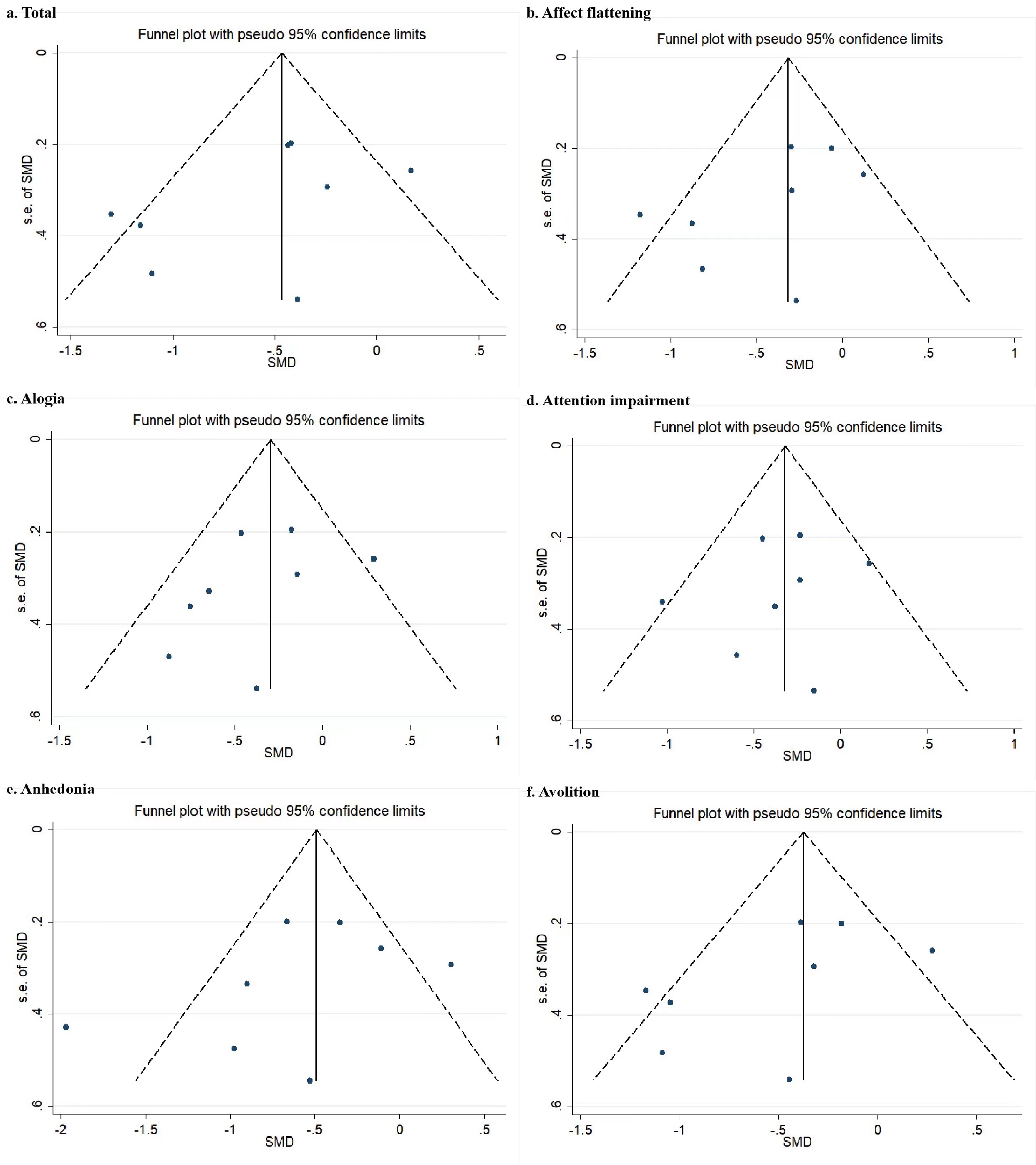


**Supplementary Fig. 1.** Funnel Plot of publication bias for NIBS for schizophrenic negative symptoms.

**Abbreviation:** s.e.: standard error; SMD: standardized mean differences; NIBS: Non-invasive brain stimulation interventions.
