# Supplementary material for: Is it more effective for anhedonia and avolition? A systematic review and meta‐analysis of non‐invasive brain stimulation interventions for negative symptoms in schizophrenia
Source: CNS Neurosci Ther. 2024 Mar 3;30(3):e14645. doi: 10.1111/cns.14645 (PMC10909625; doi:10.1111/cns.14645)
Supplement: Supplementary file 2 — Table S1–S4 [file CNS-30-e14645-s001.doc]

**Supplementary Table 1**

PRISMA 2020 checklist of current systematic review and meta-analysis.

| **Section and Topic** | **Item #** | **Checklist item** | **Location where item is reported** |
| --- | --- | --- | --- |
| TITLE | | |  |
| Title | 1 | Identify the report as a systematic review. | 1 |
| ABSTRACT | | |  |
| Abstract | 2 | See the PRISMA 2020 for Abstracts checklist. | 2-3 |
| INTRODUCTION | | |  |
| Rationale | 3 | Describe the rationale for the review in the context of existing knowledge. | 4-5 |
| Objectives | 4 | Provide an explicit statement of the objective(s) or question(s) the review addresses. | 4-5 |
| METHODS | | |  |
| Eligibility criteria | 5 | Specify the inclusion and exclusion criteria for the review and how studies were grouped for the syntheses. | 6-7 |
| Information sources | 6 | Specify all databases, registers, websites, organisations, reference lists and other sources searched or consulted to identify studies. Specify the date when each source was last searched or consulted. | 6-7, Supplementary Table 2 |
| Search strategy | 7 | Present the full search strategies for all databases, registers and websites, including any filters and limits used. | 6-7, Supplementary Table 2 |
| Selection process | 8 | Specify the methods used to decide whether a study met the inclusion criteria of the review, including how many reviewers screened each record and each report retrieved, whether they worked independently, and if applicable, details of automation tools used in the process. | 7-9 |
| Data collection process | 9 | Specify the methods used to collect data from reports, including how many reviewers collected data from each report, whether they worked independently, any processes for obtaining or confirming data from study investigators, and if applicable, details of automation tools used in the process. | 7-9 |
| Data items | 10a | List and define all outcomes for which data were sought. Specify whether all results that were compatible with each outcome domain in each study were sought (e.g. for all measures, time points, analyses), and if not, the methods used to decide which results to collect. | 7, Table1 |
|  | 10b | List and define all other variables for which data were sought (e.g. participant and intervention characteristics, funding sources). Describe any assumptions made about any missing or unclear information. | 7, Table1 |
| Study risk of bias assessment | 11 | Specify the methods used to assess risk of bias in the included studies, including details of the tool(s) used, how many reviewers assessed each study and whether they worked independently, and if applicable, details of automation tools used in the process. | 6, 7-9 |
| Effect measures | 12 | Specify for each outcome the effect measure(s) (e.g. risk ratio, mean difference) used in the synthesis or presentation of results. | 7-9 |
| Synthesis methods | 13a | Describe the processes used to decide which studies were eligible for each synthesis (e.g. tabulating the study intervention characteristics and comparing against the planned groups for each synthesis (item #5)). | 6-7 |
|  | 13b | Describe any methods required to prepare the data for presentation or synthesis, such as handling of missing summary statistics, or data conversions. | 7-9 |
|  | 13c | Describe any methods used to tabulate or visually display results of individual studies and syntheses. | 7-9 |
|  | 13d | Describe any methods used to synthesize results and provide a rationale for the choice(s). If meta-analysis was performed, describe the model(s), method(s) to identify the presence and extent of statistical heterogeneity, and software package(s) used. | 7-9 |
|  | 13e | Describe any methods used to explore possible causes of heterogeneity among study results (e.g. subgroup analysis, meta-regression). | 7-9 |
|  | 13f | Describe any sensitivity analyses conducted to assess robustness of the synthesized results. | 7-9 |
| Reporting bias assessment | 14 | Describe any methods used to assess risk of bias due to missing results in a synthesis (arising from reporting biases). | 7-9 |
| Certainty assessment | 15 | Describe any methods used to assess certainty (or confidence) in the body of evidence for an outcome. | 7-9 |
| RESULTS | | |  |
| Study selection | 16a | Describe the results of the search and selection process, from the number of records identified in the search to the number of studies included in the review, ideally using a flow diagram. | 9, Supplementary Table 1 |
|  | 16b | Cite studies that might appear to meet the inclusion criteria, but which were excluded, and explain why they were excluded. | / |
| Study characteristics | 17 | Cite each included study and present its characteristics. | 9, Table1 |
| Risk of bias in studies | 18 | Present assessments of risk of bias for each included study. | 10-12, Supplementary Table 3 |
| Results of individual studies | 19 | For all outcomes, present, for each study: (a) summary statistics for each group (where appropriate) and (b) an effect estimate and its precision (e.g. confidence/credible interval), ideally using structured tables or plots. | 9-12, Figure 2-4 |
| Results of syntheses | 20a | For each synthesis, briefly summarise the characteristics and risk of bias among contributing studies. | 9-12, Supplementary Figure 1 |
|  | 20b | Present results of all statistical syntheses conducted. If meta-analysis was done, present for each the summary estimate and its precision (e.g. confidence/credible interval) and measures of statistical heterogeneity. If comparing groups, describe the direction of the effect. | 9-12, Figure 2-4 |
|  | 20c | Present results of all investigations of possible causes of heterogeneity among study results. | 11-12 |
|  | 20d | Present results of all sensitivity analyses conducted to assess the robustness of the synthesized results. | 10-12 |
| Reporting biases | 21 | Present assessments of risk of bias due to missing results (arising from reporting biases) for each synthesis assessed. | 10-12 |
| Certainty of evidence | 22 | Present assessments of certainty (or confidence) in the body of evidence for each outcome assessed. | 12, Supplementary Table 4 |
| DISCUSSION | | |  |
| Discussion | 23a | Provide a general interpretation of the results in the context of other evidence. | 12-15 |
|  | 23b | Discuss any limitations of the evidence included in the review. | 15 |
|  | 23c | Discuss any limitations of the review processes used. | 15 |
|  | 23d | Discuss implications of the results for practice, policy, and future research. | 14-15 |
| OTHER INFORMATION | | |  |
| Registration and protocol | 24a | Provide registration information for the review, including register name and registration number, or state that the review was not registered. | 6 |
|  | 24b | Indicate where the review protocol can be accessed, or state that a protocol was not prepared. | 6 |
|  | 24c | Describe and explain any amendments to information provided at registration or in the protocol. | 6 |
| Support | 25 | Describe sources of financial or non-financial support for the review, and the role of the funders or sponsors in the review. | 17 |
| Competing interests | 26 | Declare any competing interests of review authors. | 16 |
| Availability of data, code and other materials | 27 | Report which of the following are publicly available and where they can be found: template data collection forms; data extracted from included studies; data used for all analyses; analytic code; any other materials used in the review. | 17 |

**Supplementary Table 2**

Keywords in each database and search result.

| **Database** | **Keyword** | **Filter** | **Date** | **Result** |
| --- | --- | --- | --- | --- |
| PubMed | (non-invasive brain stimulation OR noninvasive brain stimulation OR NiBS OR transcranial magnetic stimulation OR TMS OR transcranial electrical stimulation OR tES OR transcranial direct current stimulation OR tDCS OR transcranial alternating current stimulation OR tACS OR transcranial random noise stimulation OR tRNS OR theta burst stimulation OR TBS OR vagus nerve stimulation OR vagal nerve stimulation OR tVNS OR VNS OR static magnetic field stimulation OR tSMS) AND (negative symptoms OR anhedonia OR alogia OR asociality OR avolition OR apathy) AND (schizophrenia OR schizophrenic disorder) AND (random OR randomized OR randomised) | NA | 2023/10/17 | 147 |
| Web of Science | (TS=(non-invasive brain stimulation) OR TS=(noninvasive brain stimulation) OR TS=(NiBS) OR TS=(transcranial magnetic stimulation) OR TS=(TMS) OR TS=(transcranial electrical stimulation) OR TS=(tES) OR TS=(transcranial direct current stimulation) OR TS=(tDCS) OR TS=(transcranial alternating current stimulation) OR TS=(tACS) OR TS=( transcranial random noise stimulation) OR TS=(tRNS) OR TS=(theta burst stimulation) OR TS=(TBS) OR TS=( vagus nerve stimulation) OR TS=(vagal nerve stimulation) OR TS=(tVNS) OR TS=(VNS) OR TS=(static magnetic field stimulation) OR TS=(tSMS)) AND (TS=(negative symptoms) OR TS=(anhedonia) OR TS=(alogia) OR TS=(asociality) OR TS=( avolition) OR TS=(apathy)) AND (TS=(schizophrenia) OR TS=(schizophrenic disorder)) AND (TS=(random) OR TS=(randomized) OR TS=(randomised)) | NA | 2023/10/17 | 208 |
| Clinicaltrials.gov | (non-invasive brain stimulation) AND (schizophrenia) AND (negative symptom) | NA | 2023/10/17 | 8 |
| Embase | (non-invasive brain stimulation OR noninvasive brain stimulation OR NiBS OR transcranial magnetic stimulation OR TMS OR transcranial electrical stimulation OR tES OR transcranial direct current stimulation OR tDCS OR transcranial alternating current stimulation OR tACS OR transcranial random noise stimulation OR tRNS OR theta burst stimulation OR TBS OR vagus nerve stimulation OR vagal nerve stimulation OR tVNS OR VNS OR static magnetic field stimulation OR tSMS) AND (negative symptoms OR anhedonia OR alogia OR asociality OR avolition OR apathy) AND (schizophrenia OR schizophrenic disorder) AND (random OR randomized OR randomised) | NA | 2023/10/17 | 256 |
| Cochrane CENTRAL | (non-invasive brain stimulation OR noninvasive brain stimulation OR NiBS OR transcranial magnetic stimulation OR TMS OR transcranial electrical stimulation OR tES OR transcranial direct current stimulation OR tDCS OR transcranial alternating current stimulation OR tACS OR transcranial random noise stimulation OR tRNS OR theta burst stimulation OR TBS OR vagus nerve stimulation OR vagal nerve stimulation OR tVNS OR VNS OR static magnetic field stimulation OR tSMS) AND (negative symptoms OR anhedonia OR alogia OR asociality OR avolition OR apathy) AND (schizophrenia OR schizophrenic disorder) AND (random OR randomized OR randomised) | NA | 2023/10/18 | 206 |
| PsycINFO | (non-invasive brain stimulation OR noninvasive brain stimulation OR NiBS OR transcranial magnetic stimulation OR TMS OR transcranial electrical stimulation OR tES OR transcranial direct current stimulation OR tDCS OR transcranial alternating current stimulation OR tACS OR transcranial random noise stimulation OR tRNS OR theta burst stimulation OR TBS OR vagus nerve stimulation OR vagal nerve stimulation OR tVNS OR VNS OR static magnetic field stimulation OR tSMS) AND (negative symptoms OR anhedonia OR alogia OR asociality OR avolition OR apathy) AND (schizophrenia OR schizophrenic disorder) AND (random OR randomized OR randomised) | NA | 2023/10/25 | 212 |
| OpenGrey | (non-invasive brain stimulation OR noninvasive brain stimulation OR NiBS OR transcranial magnetic stimulation OR TMS OR transcranial electrical stimulation OR tES OR transcranial direct current stimulation OR tDCS OR transcranial alternating current stimulation OR tACS OR transcranial random noise stimulation OR tRNS OR theta burst stimulation OR TBS OR vagus nerve stimulation OR vagal nerve stimulation OR tVNS OR VNS OR static magnetic field stimulation OR tSMS) AND (negative symptoms OR anhedonia OR alogia OR asociality OR avolition OR apathy) AND (schizophrenia) | NA | 2023/10/27 | 12 |

Abbreviation: NA: not applied

**Supplementary Table 3**

Assessment of study quality according to PEDRo Scale.

| PEDro scale | Basavaraju  (2021) | Dharani  (2021) | Du  (2022) | Gan (2021) | Kumar (2020) | Palm  (2016) | Prikryl  (2013) | Quan  (2015) |
| --- | --- | --- | --- | --- | --- | --- | --- | --- |
| Eligibility | 1 | 1 | 1 | 1 | 1 | 1 | 1 | 1 |
| Randomization | 1 | 1 | 1 | 1 | 1 | 1 | 1 | 1 |
| Allocation | 1 | 1 | 1 | 1 | 1 | 1 | 1 | 1 |
| Groups similar | 1 | 1 | 1 | 1 | 1 | 1 | 1 | 1 |
| Subjects blinded | 1 | 1 | 1 | 1 | 1 | 1 | 1 | 1 |
| Therapists blinded | 1 | 0 | 0 | 1 | 0 | 1 | 0 | 0 |
| Raters blinded | 1 | 1 | 1 | 1 | 1 | 1 | 1 | 1 |
| Low attrition | 1 | 1 | 1 | 1 | 1 | 1 | 1 | 1 |
| ITT | 1 | 1 | 1 | 1 | 1 | 1 | 1 | 1 |
| Complete reporting | 1 | 1 | 1 | 1 | 1 | 1 | 1 | 1 |
| Complete reporting | 1 | 1 | 1 | 1 | 1 | 1 | 1 | 1 |
| PEDro total score | 11 | 10 | 10 | 11 | 10 | 11 | 10 | 10 |

**Supplementary Table 4**

Quality of evidence for outcome.

| **Outcome** | **Comparisons** | **Absolute (95% CI)** | **Number of patients (Number of studies)** | **Certainty of evidence** |
| --- | --- | --- | --- | --- |
| **Negative symptom** | NIBS active vs sham | -0.54 (-0.88, -0.21) | 431 (8) | ⨁⨁◯◯ Low^1,2^ |
| **Affect flattening** | NIBS active vs sham | -0.39 (-0.68, -0.09) | 431 (8) | ⨁⨁◯◯ Low^1,2^ |
| **Alogia** | NIBS active vs sham | -0.32 (-0.57, -0.06) | 431 (8) | ⨁⨁⨁◯ Moderate^1^ |
| **Attention impairment** | NIBS active vs sham | -0.33 (-0.56, -0.10) | 431 (8) | ⨁⨁⨁◯ Moderate^1^ |
| **Anhedonia** | NIBS active vs sham | -0.63 (-0.98, -0.28) | 431 (8) | ⨁⨁◯◯ Low^1,2^ |
| **Avolition** | NIBS active vs sham | -0.47 (-0.81, -0.13) | 431 (8) | ⨁⨁◯◯ Low^1,2^ |

**Ratings:** High quality—We are very confident that the true effect lies close to that of the estimate of the effect; Moderate quality—We are moderately confident in the effect estimate: the true effect is likely to be close to the estimate of the effect, but there is a possibility that it is substantially different; Low quality—Our confidence in the effect estimate is limited: the true effect may be substantially different from the estimate of the effect; Very low quality—We have very little confidence in the effect estimate: the true effect is likely to be substantially different from the estimate of effect.

^1^ Imprecision is reflected that the number of participants is less than 400 for each outcome.

^2^ Heterogeneity is reflected in the heterogeneity test results showing I^2^>50% and P<0.1.

**Abbreviation:** 95% CI: 95% confidence interval; NIBS: Non-invasive brain stimulation interventions.
